# Low expression levels of hepsin and TMPRSS3 are associated with poor breast cancer survival

**DOI:** 10.1186/s12885-015-1440-5

**Published:** 2015-05-27

**Authors:** Mikko Pelkonen, Kaisa Luostari, Maria Tengström, Hermanni Ahonen, Bozena Berdel, Vesa Kataja, Ylermi Soini, Veli-Matti Kosma, Arto Mannermaa

**Affiliations:** 1 Institute of Clinical Medicine, Pathology and Forensic Medicine, University of Eastern Finland, P.O. Box 1627, FI-70211 Kuopio, Finland; 2 Biocenter Kuopio and Cancer Center of Eastern Finland, University of Eastern Finland, P.O. Box 1627, FI-70211 Kuopio, Finland; 3 Imaging Center, Clinical Pathology, Kuopio University Hospital, P.O. Box 1777, FI-70211 Kuopio, Finland; 4 Institute of Clinical Medicine, Oncology, University of Eastern Finland, P.O. Box 1627, FI-70211 Kuopio, Finland; 5 Cancer Center, Kuopio University Hospital, P.O. Box 1777, FI-70211 Kuopio, Finland

**Keywords:** Biomarkers, Breast cancer, Extracellular matrix, Gene expression profiling, Hepsin, Membrane-associated proteins, Prognosis, TMPRSS1, Type II transmembrane serine proteases, TMPRSS3

## Abstract

**Background:**

Hepsin, (also called TMPRSS1) and TMPRSS3 are type II transmembrane serine proteases (TTSPs) that are involved in cancer progression. TTSPs can remodel extracellular matrix (ECM) and, when dysregulated, promote tumor progression and metastasis by inducing defects in basement membrane and ECM molecules. This study investigated whether the gene and protein expression levels of these TTSPs were associated with breast cancer characteristics or survival.

**Methods:**

Immunohistochemical staining was used to evaluate hepsin levels in 372 breast cancer samples and TMPRSS3 levels in 373 samples. *TMPRSS1* mRNA expression was determined in 125 invasive and 16 benign breast tumor samples, and *TMPRSS3* mRNA expression was determined in 167 invasive and 23 benign breast tumor samples. The gene and protein expression levels were analyzed for associations with breast cancer-specific survival and clinicopathological parameters.

**Results:**

Low *TMPRSS1* and *TMPRSS3* mRNA expression levels were independent prognostic factors for poor breast cancer survival during the 20-year follow-up (*TMPRSS1*, *P* = 0.023; HR, 2.065; 95 % CI, 1.106–3.856; *TMPRSS3*, *P* = 0.013; HR, 2.106; 95 % CI, 1.167–3.800). Low expression of the two genes at the mRNA and protein levels associated with poorer survival compared to high levels (log rank *P*-values 0.015–0.042). Low *TMPRSS1* mRNA expression was also an independent marker of poor breast cancer prognosis in patients treated with radiotherapy (*P* = 0.034; HR, 2.344; 95 % CI, 1.065–5.160). Grade III tumors, large tumor size, and metastasis were associated with low mRNA and protein expression levels.

**Conclusions:**

The results suggest that the TTSPs hepsin and TMPRSS3 may have similar biological functions in the molecular pathology of breast cancer. Low mRNA and protein expression levels of the studied TTSPs were prognostic markers of poor survival in breast cancer.

**Electronic supplementary material:**

The online version of this article (doi:10.1186/s12885-015-1440-5) contains supplementary material, which is available to authorized users.

## Background

Globally, breast cancer is the most commonly diagnosed cancer in women, while metastatic disease is the leading cause of cancer-related deaths in this group [1]. Epithelial integrity and intact extracellular matrix (ECM), which includes basement membrane and interstitial connectivity tissue, are essential for normal cell behavior and tissue homeostasis [2]. Remodeling and degradation of the ECM, along with defects in structural cell-adhesion molecules, play a significant role in breast cancer progression [3]. Type II transmembrane serine proteases (TTSPs) are a relatively new subfamily of S1 class serine proteases in humans comprised of 17 proteolytic enzymes [4, 5]. In addition to their roles in normal tissue development and homeostasis, TTSPs are also involved in several human diseases, including cancer, and many show potential as biomarkers of tumor progression and represent prospective therapeutic targets [6, 7]. TTSPs localize to the cell membrane and are able to degrade the ECM and remodel intercellular and cell-ECM junctions. Accordingly, dysregulation of TTSPs is thought to be involved in the early stages of tumorigenesis, tumor growth, and cancer cell invasiveness that lead to metastasis [8, 9]. In this study, we looked at the expression of two members of the TTSP family, hepsin (also called TMPRSS1), which is encoded by the *TMPRSS1* gene, and TMPRSS3, encoded by the *TMPRSS3* gene.

Hepsin upregulation in malignant tumors has been demonstrated in prostate and ovarian cancers as well as in renal cell carcinoma [10–13]. A recent study used immunohistochemistry to show that hepsin protein levels were upregulated in human breast cancer tumor samples [14]. *TMPRSS1* mRNA overexpression is associated with ER(α)-positive human breast tumors [15], while *TMPRSS3* overexpression has been implicated in pancreatic and epithelial ovarian cancers [16, 17]. Missense mutations in the *TMPRSS3* gene that lead to structural TMPRSS3 defects are associated with hereditary deafness [18]. Both hepsin and TMPRSS3 belong to the hepsin/TMPRSS subfamily of TTSPs and share structural features [5, 6]. TTSPs are anchored to the cell membrane via an N-terminal transmembrane domain. At the C-terminus, TTSPs have an extracellular serine protease domain that is required for their catalytic activity [4, 7]. Notably, several soluble forms of TTSPs that retain catalytic activity have also been detected [4, 9]. Hepsin and TMPRSS3 appear to be capable of autocatalytic activation, suggesting that they play roles as initiators of proteolytic cascades that lead to ECM remodeling [19, 20]. Overexpressed hepsin activates proteolytic pathways and also directly interferes with cell-cell and cell-ECM adhesion molecules. Hepsin can activate hepatocyte growth factor (HGF) and urokinase-type plasminogen activator- (uPA) mediated proteolytic pathways, which results in ECM degradation [21–23]. Hepsin plays a physiological role as it directly and specifically cleaves laminin-332 (ln-332, previously termed laminin-5), an important ECM protein involved in maintaining the structural integrity of the basement membrane [24]. It was shown recently that hepsin becomes mislocalized when liver kinase B1 (lkb1) expression is lost and that overexpressed hepsin induces basement membrane degradation in breast cancer [25].

This is the first study to examine *TMPRSS3* gene expression in a set of clinical breast cancer samples and to investigate whether altered *TMPRSS1* and *TMPRSS3* gene expression has an impact on the clinical outcome of breast cancer patients. Here, we analyzed the associations of mRNA and protein expression of these genes with clinicopathological parameters and breast cancer-specific survival. Recently, we reported that *TMPRSS3* SNP rs3814903 associated with both breast cancer risk and survival and SNP rs11203200 associated with breast cancer survival [26]. Furthermore, *TMPRSS1* SNPs rs12151195 and rs12461158 remained independent prognostic factors of breast cancer survival [26]. Our previous study showed that another member of the TTSP family, matriptase (encoded by the *ST14* gene), is associated with breast cancer survival [27]. We also showed that several *TMPRSS6* (encoding matriptase-2) variants are related to breast cancer prognosis and matriptase-2 expression levels decrease with tumor progression [28]. These previous findings prompted us to investigate whether altered expression of hepsin and TMPRSS3 might also have a role in the molecular pathology of breast cancer. Although the physiological substrates for TMPRSS3 remain unclear, it is possible that the biological mechanisms that lead to ECM degradation are similar to those of hepsin. When overexpressed in breast cancer, hepsin and TMPRSS3 could promote cancer cell invasiveness via dysregulated proteolytic activity. This results in defects in the basement membrane and in uncontrolled ECM degradation. However, the expression levels seem to decrease as tumor malignancy increases, and low expression levels of these proteins are associated with poor breast cancer survival as well as with the adjuvant treatments the patients received.

## Methods

### Patients

The patient samples used in this study were obtained from the Kuopio Breast Cancer Project (KBCP) sample set, which includes 497 potential breast cancer cases from the Northern Savo region of Eastern Finland. The patients were diagnosed at Kuopio University Hospital between April 1990 and December 1995 [27, 29]. All the patients are of Caucasian race. The KBCP, including this study, was approved by the official Research Ethics Committee of Hospital District of Northern Savo. Informed written consents were obtained from all of the patients and this study was carried out in compliance with the Declaration of Helsinki. Patient follow-up status was last revised in February 2011. Table 1 shows the clinicopathological characteristics of the breast tumor cases in this study as well as data on the adjuvant treatments the patients received.

**Table 1:** Clinicopathological charasteristics of the patients

	Cases in *TMPRSS1*		Cases in *TMPRSS3*		Cases in TMPRSS1*		Cases in TMPRSS3	
	mRNA expression		mRNA expression		protein expression		protein expression	
Clinical variable	n	%	n	%	n	%	n	%
Breast tumor samples								
Malignant	125	88.7	167	87.9	372	100	373	100
Benign	16	11.3	23	12.1				
Histological type								
Ductal	89	63.2	118	62.1	236	63.5	237	63.5
Lobular	20	14.2	31	16.3	73	19.6	72	19.3
Other malignant	16	11.3	18	9.5	63	16.9	64	17.2
Benign	16	11.3	23	12.1				
Age at diagnosis								
< = 39	12	9.6	14	8.4	31	8.3	31	8.3
40–49	22	17.6	29	17.4	91	24.5	91	24.4
50–59	29	23.2	38	22.8	88	23.7	88	23.6
60–69	18	14.4	28	16.7	53	14.2	54	14.5
> = 70	44	35.2	58	34.7	109	29.3	109	29.2
Tumor grade								
I	24	19.2	28	16.8	87	23.4	88	23.6
II	51	40.8	74	44.3	162	43.6	161	43.2
III	48	38.4	60	35.9	105	28.2	106	28.4
NA	2	1.6	5	3.0	18	4.8	18	4.8
Tumor size								
T1	46	36.8	63	37.7	173	46.5	174	46.6
T2	64	51.2	84	50.3	160	43.0	160	42.9
T3	9	7.2	12	7.2	19	5.1	20	5.4
T4	6	4.8	8	4.8	15	4.0	15	4.0
NA					5	1.4	4	1.1
Nodal status								
Negative	71	56.8	87	52.1	197	53.0	198	53.1
Positive	51	40.8	73	43.7	155	41.6	155	41.5
NA	3	2.4	7	4.2	20	5.4	20	5.4
Stage								
I	34	27.2	42	25.1	121	32.5	122	32.7
II	74	59.2	97	58.1	184	49.5	184	49.3
III	10	8.0	14	8.4	32	8.6	32	8.6
IV	4	3.2	7	4.2	13	3.5	13	3.5
NA	3	2.4	7	4.2	22	5.9	22	5.9
ER status								
Negative	40	32.0	50	29.9	82	22.0	83	22.2
Positive	83	66.4	111	66.5	270	72.6	270	72.4
NA	2	1.6	6	3.6	20	5.4	20	5.4
PR status								
Negative	56	44.8	70	41.9	140	37.6	141	37.8
Positive	67	53.6	91	54.5	212	57.0	211	56.6
NA	2	1.6	6	3.6	20	5.4	21	5.6
HER2 status								
Negative	99	79.2	130	77.8	299	80.4	299	80.2
Positive	19	15.2	24	14.4	46	12.3	46	12.3
NA	7	5.6	13	7.8	27	7.3	28	7.5
ER/PR/HER2 status								
Triple-negative	24	19.2	29	17.4	42	11.3	43	11.5
Non-triple-negative	94	75.2	122	73.0	289	77.7	288	77.2
NA	7	5.6	16	9.6	41	11.0	42	11.3
Radiotherapy								
Yes	66	52.8	84	50.3	205	55.1	208	55.8
No	59	47.2	83	49.7	167	44.9	165	44.2
Chemotherapy								
Yes	18	14.4	26	15.6	69	18.5	69	18.5
No	107	85.6	141	84.4	303	81.5	304	81.5
Tamoxifen								
Yes	23	18.4	31	18.6	62	16.7	62	16.6
No	102	81.6	136	81.4	310	83.3	311	83.4
Latest follow-up status								
Alive	38	30.4	52	31.2	149	40.1	149	39.9
Died of breast cancer	42	33.6	56	33.5	112	30.1	113	30.3
Died of other reason	45	36.0	59	35.3	111	29.8	111	29.8

NA, data not avalaible; *hepsin

### RNA extraction and cDNA synthesis

Human breast tumor tissue sample retrieval during surgery, RNA extraction from the tumor samples and cDNA synthesis were performed as described in Kauppinen et al. [27]. The *mir*Vana™ miRNA Isolation Kit (Life Technologies, Carlsbad, CA) was used to extract total RNA from frozen tissue samples, and the High Capacity cDNA Reverse Transcription Kit (Life Technologies) for cDNA synthesis. RNA extraction and cDNA synthesis were performed according to the manufacturer’s instructions.

### Quantitative real-time PCR

Of the KBCP sample set, 125 invasive breast cancer samples and 16 benign breast tumor samples were available for *TMPRSS1* mRNA absolute quantification by real-time PCR, and 167 invasive and 23 benign samples were available for *TMPRSS3* mRNA quantification. TaqMan Gene Expression Assays (Life Technologies) were used according to the manufacturer’s instructions (assay #Hs01056332_m1 for *TMPRSS1* and #Hs00225161_m1 for *TMPRSS3*), and peptidylprolyl isomerase A (*PPIA*) was used as an endogenous control [Human Cyc Pre-Developed TaqMan Assay Reagents (20X), Life Technologies] [30]. Brilliant III Ultra-Fast QPCR Master Mix (Agilent Technologies, Santa Clara, CA) and Mx3000P real-time PCR system with MxPro-Mx3000P v4.10 software (Agilent Technologies) were used according to the manufacturer’s instructions. The PCR thermal profile was 1 cycle at 95-°C for 3 min followed by 45-55 cycles at 95-°C for 20 s plus 30 s at 60-°C. The assays for the studied gene and the control were in the same reaction. Samples were analyzed in triplicate in 96-well plates. The amount of cDNA varied from 2-75 ng in a final volume of 20-μl, and each plate included standard curves for sample quantification using a serial dilution of cDNA that was synthesized from 5 randomly-selected KBCP tumor samples. The relative mRNA expression values were calculated by dividing the raw expression of the studied gene with the raw *PPIA* expression in the sample.

### Immunohistochemistry

For immunohistochemical staining, 372 invasive breast cancer tumor samples were available for hepsin analysis and 373 samples were available for TMPRSS3 analysis. Immunohistochemical staining was performed on 4-μm sections cut from the tissue microarray (TMA) blocks. The TMA blocks were constructed with a custom-built instrument (Beecher Instruments, Silver Spring, MD). The sample diameter of the tissue core in the array block was 1000 μm and three samples from tumor tissue of each case were studied. After deparaffinization and rehydration, the sections for TMPRSS3 analysis were heated in a microwave oven for 3 × 5 min in citrate buffer (pH 6.0). The sections for hepsin analysis were not heated. The slides were treated for 5 min with 5 % hydrogen peroxide to block endogenous peroxidase, then incubated for 35 min at room temperature in 1.5 % normal serum diluted in PBS to block non-specific binding. The blocked sections were incubated overnight at 4-°C with the rabbit polyclonal primary antibody against hepsin (LS-C24203/28374; LifeSpan BioSciences, Seattle, WA) at a dilution of 1:250 or with an antibody against TMPRSS3 (NBP1-19582; Novus Biologicals, Littleton, CO) at a dilution of 1:250. The slides were then incubated with a biotinylated secondary antibody for 35 min and with an avidin-biotin-peroxidase complex for 45 min [Vectastain Elite ABC Kit (anti-rabbit IgG); Vector Laboratories, Burlingame, CA]. Slides were rinsed with PBS after each step of the immunostaining procedure. The color was developed using diaminobenzidine tetrahydrochloride (DAB; Sigma, St. Louis, MO). The slides were counterstained with Mayer's hematoxylin, washed, dehydrated, cleared, and mounted with Depex (BDH, Poole, UK). For the negative controls, the primary antibody was omitted.

**Fig. 1:**
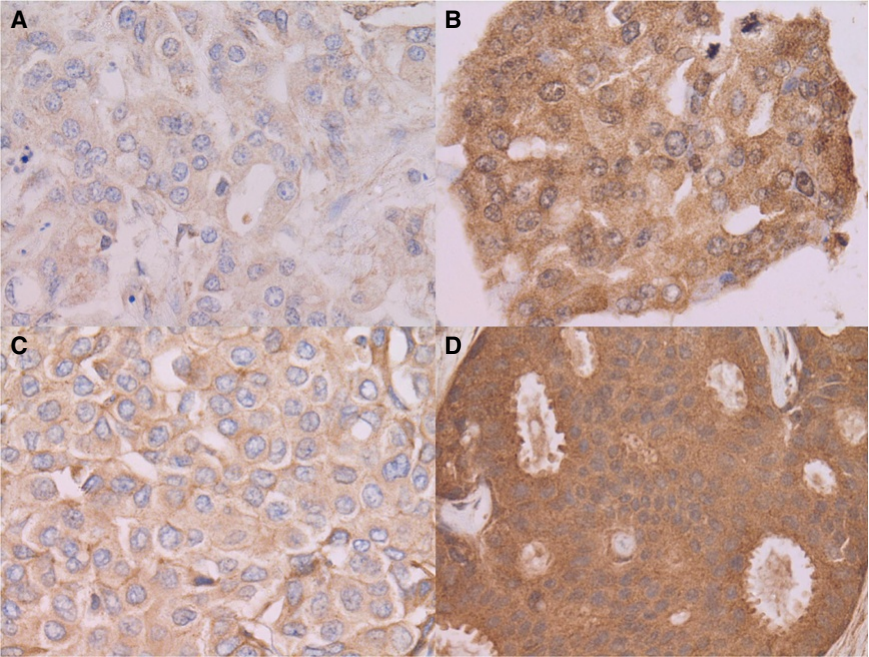
Immunohistochemical staining of hepsin and TMPRSS3 in invasive ductal breast cancer. Cytoplasmic immunostaining of epithelial tumor cells: **a**, weak staining of hepsin (score of 1 for intensity); **b**, intense staining of hepsin (score of 3); **c**, weak staining of TMPRSS3 (score of 1); **d**, intense staining of TMPRSS3 (score of 3). All panels, 400x magnification

Three slides from each TMA block were examined in triplicate by two researchers (BB, HA) under the supervision of a senior pathologist (YS). The immunoreactivity of hepsin and TMPRSS3 in the cytoplasm of epithelial tumor cells was analyzed, and the intensity and the extent of staining were scored (0, negative; 1, weak; 2, moderate; 3, intense; Fig. 1a-d). The three slides were evaluated separately by researchers and were re-evaluated when values were inconsistent to achieve a consensus. The tumor samples were divided into low and high expression groups according to the median value of immunohistochemical staining scores.

### Statistical analysis

Statistical analyses were performed using IBM SPSS Statistics 19 software (IBM Corporation, Armonk, NY). The non-parametric Mann-Whitney U test and the Kruskal-Wallis test were used to study differences in continuous mRNA expression values according to different clinicopathological parameters. Fisher’s exact test was used to study associations between protein expression and clinicopathological parameters. The odds ratios (ORs) and the 95 % confidence intervals (95 % CIs) were determined using logistic regression analysis to describe the strength of statistically significant associations between expression levels and clinicopathological characteristics. The Kaplan-Meier method was used in univariate survival analyses. Multivariate Cox’s proportional hazards analysis was carried out in a forward stepwise method to estimate the hazard factors having an impact on breast cancer-specific death and relapse. In addition to mRNA and protein expressions, Cox regression analysis examined the following clinicopathological parameters: tumor grade, nodal status, tumor size, estrogen receptor (ER) status, progesterone receptor (PR) status, and tumor histologic type. In addition, the adjuvant treatments were used as variables in the analyses including the treatment data. All statistical tests were two-sided, and a *P* value of 0.05 was considered statistically significant.

We used an online Kaplan-Meier survival analysis tool to validate the value of *TMPRSS1* and *TMPRSS3* as prognostic biomarkers in breast cancer (http://kmplot.com/analysis/index.php?p=service&cancer=breast) [31]. The Kaplan-Meier plotter uses gene expression data and relapse-free and overall survival information which are downloaded from GEO (Affymetrix microarrays only), EGA and TCGA. The patient samples are divided into two groups according to the median gene expression value similar to our analysis method. The groups are then compared by Kaplan-Meier plot and the hazard ratio with 95 % confidence intervals and log rank *P* values are calculated [31].

## Results

### Low mRNA expression and low protein expression are associated with advanced breast cancer tumor malignancy

The results of quantitative real-time PCR and immunohistochemical staining were analyzed for associations with the clinicopathological parameters of each patient. Table 2 presents the statistical association results for *TMPRSS1* and *TMPRSS3* mRNA expression, and Additional file 1: Table S1 presents the statistical association results for hepsin and TMPRSS3 protein expression. *TMPRSS1* and *TMPRSS3* mRNA expression was high in well-differentiated malignant breast tumors compared to benign breast tumors (Table 2; Additional file 2: Figure S1A-B). However, poorly differentiated tumors expressed low mRNA levels of both genes (*TMPRSS1*: *P* = 0.000015 and *TMPRSS3*: *P* = 0.0002; Kruskal-Wallis test; Table 2; Additional file 2: Figure S1A-B). Likewise, logistic regression analysis showed that low hepsin expression levels were associated with poorly differentiated tumors (*P* = 0.00009; OR, 3.289; 95 % CI, 1.811-5.973; Additional file 1: Table S1), as were low levels of TMPRSS3 protein expression (*P* = 0.0000002; OR, 5.006; 95 % CI, 2.721-9.209; Additional file 1: Table S1).

**Table 2:** Significant clinical variables associated with *TMPRSS1* and *TMPRSS3* mRNA expression

	*TMPRSS1* expression^a^			*TMPRSS3* expression^a^		
Clinical variable	*n* (%)	*P*	Median / IQR	*n* (%)	*P*	Median / IQR
Tumor type		0.002			NS	
Benign	16 (11.3)		0.27 / 0.57	23 (12.1)		0.98 / 0.56
Malignant	125 (88.7)		0.96 / 2.10	167 (87.9)		0.92 / 1.74
Tumor grade		0.000015			0.0002	
I	24 (19.5)	0.000007^b^	2.03 / 2.74	28 (17.3)	0.016^b^	1.76 / 2.80
II	51 (41.5)	NS^c^	1.67 / 2.41	74 (45.7)	NS^c^	1.29 / 2.03
III	48 (39.0)	0.000004^d^	0.55 / 0.88	60 (37.0)	0.0001^d^	0.55 / 0.84
ER status		0.000003			0.0027	
Negative	40 (32.5)		0.45 / 0.84	50 (31.1)		0.55 / 0.84
Positive	83 (67.5)		1.67 / 2.44	111 (68.9)		1.24 / 2.34
PR status		0.001			0.0076	
Negative	56 (45.5)		0.59 / 1.39	70 (43.5)		0.67 / 1.21
Positive	67 (54.5)		1.53 / 2.57	91 (56.5)		1.32 / 2.41
HER2 status		0.001			0.017	
Negative	99 (83.9)		1.12 / 2.25	130 (84.4)		1.08 / 2.46
Positive	19 (16.1)		0.35 / 0.75	24 (15.6)		0.58 / 0.89
ER/PR/HER2 status		0.001			NS	
Triple-negative	24 (20.3)		0.49 / 0.98	29 (19.2)		0.69 / 1.86
Non-triple-negative	94 (79.7)		1.12 / 2.33	122 (80.8)		1.09 / 1.81

IQR, Interquartile range; NS, Not significant

^a^Mann-Whitney U test was used for subgroups of two variables and Kruskal-Wallis test for subgroups of several variables

^b^*P* value for comparing mRNA expression in benign tumors versus grade I tumors

^c^*P* value for comparing mRNA expression in grade I tumors versus grade II tumors

^d^*P* value for comparing mRNA expression in grade I tumors versus grade III tumors

The results in Table 2 and Additional file 1: Table S1 show that mRNA and protein expression levels were high in well-differentiated tumors and low in poorly differentiated tumors. Furthermore, logistic regression analysis showed that low hepsin and TMPRSS3 protein expression levels were positively associated with advanced clinical stages III and IV (hepsin: *P* = 0.005; OR, 2.757; 95 % CI, 1.354-5.611 and TMPRSS3: *P* = 0.028; OR, 2.176; 95 % CI, 1.086-4.361) and that low hepsin expression was positively associated with larger tumor sizes (T3 and T4; *P* = 0.034; OR, 2.266; 95 % CI, 1.065-4.82), which indicates more extensive disease. The Mann-Whitney U test showed that low *TMPRSS1* and *TMPRSS3* expression levels were associated with ER-negative status, PR-negative status, and HER2-positive status (Table 2). In addition, low *TMPRSS1* mRNA expression was associated with triple-negative tumors (Table 2). As shown by the logistic regression analysis, low hepsin protein expression associated with positive nodal status, while low TMPRSS3 protein expression with PR-negative status and triple-negative tumors (Additional file 1: Table S1).

### Low mRNA and protein expression levels predict poor breast cancer survival

**Fig. 2:**
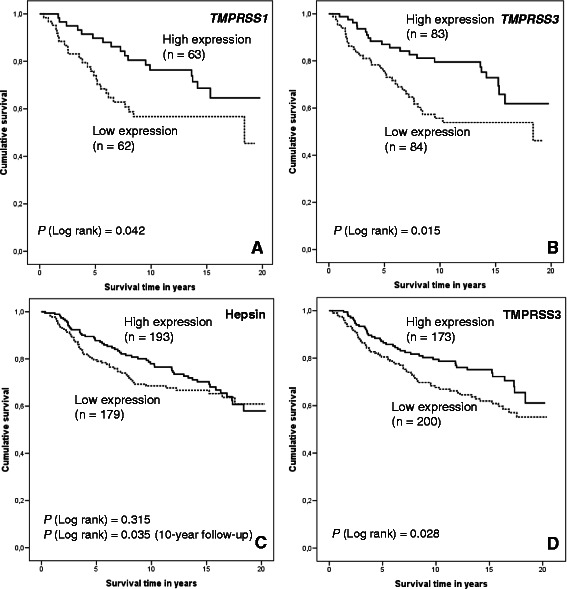
Kaplan-Meier survival analysis of the breast cancer patients according to mRNA and protein expression levels. Patients were divided into high and low expression groups relative to the median expression values. Expression of **a**, *TMPRSS1* mRNA (median follow-up time 9.84 years); **b**, *TMPRSS3* mRNA (median follow-up time 9.54 years); **c**, hepsin protein (median follow-up time 11.05 years); and **d**, TMPRSS3 protein (median follow-up time 10.94 years)

Statistical analysis of 20-year follow-up data revealed that the mRNA and protein expression levels of the studied genes had prognostic value for the breast cancer patients in this study. The univariate Kaplan-Meier analysis showed that low mRNA expression of *TMPRSS1* (log rank, *P* = 0.042; Fig. 2a) and *TMPRSS3* (log rank, *P* = 0.015; Fig. 2b) predicted poorer breast cancer-specific survival compared to high expression, as did low expression of the TMPRSS3 protein (log rank, *P* = 0.028; Fig. 2d) during the 20-year follow-up period. Similarly, low protein expression of hepsin (log rank, *P* = 0.035, Fig. 2c) predicted poorer breast cancer-specific survival during the 10-year follow-up period, yet was not significant during the 20-year follow-up period (*P* = 0.315, Fig. 2c).

**Fig. 3:**
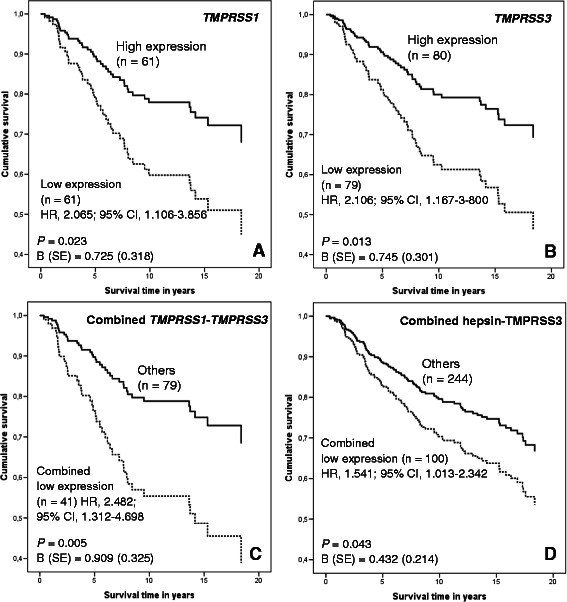
Cox regression multivariate analysis of breast cancer survival. Patients were divided into high and low expression groups relative to the median expression values (**a**, **b**). Cox regression analysis of survival according to the expression of (**a**), *TMPRSS1* mRNA (median follow-up time 9.79 years); **b**, *TMPRSS3* mRNA (median follow-up time 9.51 years); **c**, *TMPRSS1* and *TMPRSS3* mRNA (median follow-up time 9.79 years); and **d**, hepsin and TMPRSS3 protein expression (median follow-up time 11.05 years). In addition to expression levels, tumor grade, nodal status, tumor size, hormone receptor status, and histologic type were included in the multivariate analyses. Positive nodal status and large tumor size (T3, T4) were other parameters that were significantly associated with poorer breast cancer survival in the multivariate analyses

In the multivariate Cox regression survival analysis, low mRNA expression of *TMPRSS1* (*P* = 0.023; HR, 2.065; 95 % CI, 1.106-3.856; Table 3; Fig. 3a) and *TMPRSS3* (*P* = 0.013; HR, 2.106; 95 % CI, 1.167-3.800; Table 3; Fig. 3b) remained independent factors for predicting poor breast cancer survival. The clinicopathological parameters that remained independent prognostic factors of poor survival included positive nodal status and large tumor size (T3, T4) when *TMPRSS1* expression was studied in the multivariate survival analysis. In the multivariate survival analysis of *TMPRSS3* expression, ER-negative status and lobular histology were independent prognostic factors in addition to positive nodal status and large tumor size. Positive nodal status and large tumor size were statistically more significant than low mRNA expression levels in terms of poor breast cancer prognosis. Associations between protein expression and breast cancer prognosis could not be identified in the multivariate survival analysis (data not shown).

**Table 3:** Multivariate Cox regression analyses of clinicopathological variables, mRNA and protein expression levels, and breast cancer survival

Variable	B (SE)	Wald	RR (95 % CI)	*P*
Multivariate survival analysis with *TMPRSS1* mRNA expression				
Nodal status				
Negative		Ref.		
Positive	0.786 (0.339)	5.369	2.194 (1.129–4.265)	0.020
Tumor size				
T1		Ref.		
T2	0.678 (0.421)	2.597	1.970 (0.864–4.492)	0.107
T3, T4	1.385 (0.504)	7.563	3.997 (1.489–10.729)	0.006
*TMPRSS1* mRNA expression*				
Low	0.725 (0.318)	5.187	2.065 (1.106–3.856)	0.023
High		Ref.		
Multivariate survival analysis with *TMPRSS3* mRNA expression				
Nodal status				
Negative		Ref.		
Positive	1.091 (0.320)	11.596	2.976 (1.589–5.575)	0.001
Tumor size				
T1		Ref.		
T2	0.317 (0.373)	0.769	1.386 (0.668–2.874)	0.380
T3, T4	1.256 (0.452)	7.720	3.511 (1.448–8.516)	0.005
*TMPRSS3* mRNA expression*				
Low	0.745 (0.301)	6.111	2.106 (1.167–3.800)	0.013
High		Ref.		
Histology				
Ductal	0.472 (0.551)	0.734	1.603 (0.545–4.715)	0.391
Lobular	1.501 (0.589)	6.497	4.487 (1.415–14.231)	0.011
Medullary, others		Ref.		
ER status				
Negative	0.628 (0.310)	4.109	1.873 (1.021–3.437)	0.043
Positive		Ref.		
Multivariate survival analysis with combined *TMPRSS1-TMPRSS3* mRNA expression				
Combined mRNA expression^†^				
Low	0.909 (0.325)	7.803	2.482 (1.312–4.698)	0.005
Others		Ref.		
Nodal status				
Negative		Ref.		
Positive	0.975 (0.360)	7.327	2.650 (1.309–5.368)	0.007
Tumor size				
T1		Ref.		
T2	0.481 (0.433)	1.233	1.617 (0.692–3.779)	0.267
T3, T4	1.281 (0.505)	6.427	3.600 (1.337–9.691)	0.011
Multivariate survival analysis with combined hepsin-TMPRSS3 protein expression				
Nodal status				
Negative		Ref.		
Positive	0.905 (0.227)	15.942	2.473 (1.586–3.857)	0.00007
Tumor size				
T1		Ref.		
T2	0.176 (0.235)	0.559	1.192 (0.752–1.888)	0.455
T3, T4	0.891 (0.321)	7.696	2.438 (1.299–4.576)	0.006
Histology				
Ductal	0.255 (0.322)	0.628	1.291 (0.687–2.426)	0.428
Lobular	0.903 (0.343)	6.909	2.466 (1.258–4.834)	0.009
Medullary, others		Ref.		
Tumor grade				
I		Ref.		
II	0.711 (0.290)	6.025	2.036 (1.154–3.591)	0.014
III	0.532 (0.337)	2.493	1.702 (0.880–3.294)	0.114
Combined protein expression^†^				
Low	0.432 (0.214)	4.087	1.541 (1.013–2.342)	0.043
Others		Ref.		

Note: Analyses included tumor grade, histology, tumor size, nodal status, ER and PR status

B (SE), B coefficient with standard error; HR (95 % CI), hazard ratio of breast cancer death with a 95 % confidence interval; Ref., reference category in the multivariate analysis

*The relative median value of mRNA expression level was used in the analyses

^†^The ‘combined low group’ included all cases with low expression levels of both genes

Associations between relapse-free survival during the 20-year follow-up period and expression levels were studied using univariate Kaplan-Meier analysis. Patients with low levels of *TMPRSS3* mRNA and low levels of TMPRSS3 protein had more frequent relapses (*TMPRSS3*: log rank, *P* = 0.009; Additional file 3: Figure S2A and TMPRSS3: log rank, *P* = 0.003; Additional file 3: Figure S2B). In the Cox regression multivariate analysis, both low *TMPRSS3* mRNA expression and low TMPRSS3 protein expression remained independent factors that had an effect on relapse occurrence (Additional file 3: Figure S2C-D). When studied separately, the association of local recurrence with expression levels was not as strong as the association of distant recurrence with expression levels (data not shown). During 20-year follow-up period, distant metastasis occurred more frequently in patients with low *TMPRSS1* expression levels (Additional file 4: Figure S3A), low *TMPRSS3* expression levels (Additional file 4: Figure S3B), and low TMPRSS3 protein expression levels (Additional file 4: Figure S3C).

### Low *TMPRSS1* mRNA expression is associated with poor survival in patients treated with radiotherapy

**Fig. 4:**
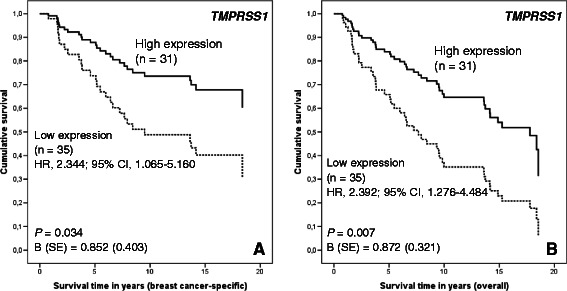
Low *TMPRSS1* mRNA expression is associated with poor survival in patients treated with radiotherapy in Cox regression multivariate analysis. Cox regression analysis of **a**, breast cancer-specific survival and **b**, overall survival according to the *TMPRSS1* mRNA expression in patients treated with radiotherapy. Adjustments were made for age, stage, grade, histologic type, hormone receptor status, hormonal treatment, and chemotherapy

Associations between the studied expression levels and breast cancer-specific and overall survival according to the adjuvant therapies given to the patients were analyzed using univariate Kaplan-Meier analysis and multivariate Cox regression survival analysis. Low *TMPRSS1* mRNA expression was associated with both poor breast cancer-specific survival (log rank, *P* = 0.030; Cox regression analysis, *P* = 0.034; HR, 2.344; 95 % CI, 1.065-5.160; Fig. 4a) and poor overall survival (log rank, *P* = 0.006; Cox regression analysis, *P* = 0.007; HR, 2.392; 95 % CI, 1.276-4.484; Fig. 4b) in patients who were treated with radiotherapy. There were no significant survival differences according to the *TMPRSS1* mRNA level in patients who did not receive radiotherapy. Furthermore, the Kaplan-Meier estimates of patients who received chemotherapy showed that low *TMPRSS1* mRNA expression was associated with poor breast cancer-specific survival (log rank, *P* = 0.028) and poor overall survival (log rank, *P* = 0.028).

When the treatment data was included in the survival analyses, low *TMPRSS3* mRNA expression was associated with poor breast cancer-specific survival (log rank, *P* = 0.039) in all the treated patients and remained an independent factor predicting more frequent relapse occurrence (log rank, *P* = 0.023; Cox regression analysis, *P* = 0.021; HR, 1.831; 95 % CI, 1.094–3.063). Low TMPRSS3 protein expression also predicted poorer relapse-free survival (log rank, *P* = 0.011; Cox regression, *P* = 0.031; HR, 1.520; 95 % CI, 1.040–2.221) compared with high TMPRSS3 expression. No significant results were found between different treatment groups regarding TMPRSS3 expression.

### The combination of low *TMPRSS1*-*TMPRSS3* mRNA and hepsin-TMPRSS3 protein expression predicts poor breast cancer survival

The statistical associations between mRNA and protein expression levels were assessed using Spearman rank correlation coefficient. *TMPRSS1* expression levels correlated with hepsin protein expression levels (*r* = 0.18; *P* = 0.05; *n* = 112), and *TMPRSS3* expression levels correlated with TMPRSS3 protein expression levels (*r* = 0.24; *P* = 0.04; *n* = 147). Positive correlations were also found between *TMPRSS1* and *TMPRSS3* mRNA expression (*r* = 0.39; *P* = 0.000007; *n* = 123) and between hepsin and TMPRSS3 protein expression (*r* = 0.27; *P* = 0.0000001; *n* = 371). Consequently, combined *TMPRSS1* and *TMPRSS3* mRNA expression and combined hepsin and TMPRSS3 protein expression were tested for statistical association with clinicopathological parameters and breast cancer-specific survival. The combination variables were formed so that the breast cancer cases that expressed low mRNA levels of both of the studied genes formed the ‘low combined mRNA expression group’ and the remaining cases formed the other (reference) group. A similar approach was used to forming a ‘low protein expression’ group. Breast cancer cases with low combined expression were associated with clinicopathological parameters that indicate advanced tumor malignancy (Additional file 5: Table S2).

Univariate Kaplan-Meier survival analyses showed that low levels of both mRNA (log rank, *P* = 0.013; Additional file 6: Figure S4A) and protein expression (log rank, *P* = 0.001; Additional file 6: Figure S4B) indicated poorer breast cancer prognosis, with low expression levels associated with poor breast cancer survival and distant recurrence during the 20-year follow-up period (Additional file 6: Figure S4C-D). Both low mRNA (*P* = 0.005; HR, 2.482; 95 % CI, 1.312–4.698; Table 3; Fig. 3c) and low protein expression (*P* = 0.043; HR, 1.541; 95 % CI, 1.013-2.342; Table 3; Fig. 3d) remained independent factors for survival in the multivariate Cox regression analysis, as did positive nodal status and large tumor size (data not shown). Taken together, these results indicate that the protein expression levels of hepsin and TMPRSS3 correlate with the mRNA levels of *TMPRSS1* and *TMPRSS3*, respectively. Further, low expression levels of *TMPRSS1* and *TMPRSS3* mRNA and hepsin and TMPRSS3 predict advanced tumor malignancy and poorer prognosis.

### The prognostic value of low *TMPRSS1* and *TMPRSS3* mRNA expression levels in breast cancer was validated in a public gene expression dataset

The results obtained from the online Kaplan-Meier plotter analysis tool presented that both low *TMPRSS1* and *TMPRSS3* expression were significantly associated with poorer relapse-free survival (*TMPRSS1*: log rank, *P* = 0; HR, 0.61; 95 % CI, 0.55–0.69; n = 3554; Additional file 7: Figure S5A and *TMPRSS3*: log rank, *P* = 3.8e-10; HR, 0.66; 95 % CI, 0.58–0.73; n = 3554; Additional file 7: Figure S5B), overall survival (*TMPRSS1*: log rank, *P* = 0.0083; HR, 0.73; 95 % CI, 0.57–0.92; n = 1117; Additional file 7: Figure S5C and *TMPRSS3*: log rank, *P* = 0.00005; HR, 0.58; 95 % CI, 0.46–0.74; n = 1117; Additional file 7: Figure S5D), and distant metastasis-free survival in breast cancer (*TMPRSS1*: log rank, *P* = 0.000099; HR, 0.67; 95 % CI, 0.55–0.82; n = 1609; Additional file 7: Figure S5E and *TMPRSS3*: log rank, *P* = 0.0000039; HR, 0.62; 95 % CI, 0.51–0.76; n = 1609; Additional file 7: Figure S5F).

## Discussion

This is the first study to link altered *TMPRSS3* expression to breast cancer tumor progression and to show that low *TMPRSS1* and *TMPRSS3* expression, both at the mRNA and protein levels, has prognostic value for poorer survival of breast cancer patients. Importantly, this is also the first cancer study to show that altered *TMPRSS3* expression has prognostic value for cancer-related death. In benign breast tumor cells, the expression levels of *TMPRSS1* and *TMPRSS3* are consistently low, whereas the expression levels are higher in cancer cells. In malignant samples, there was clearly a high degree of intertumor variation in the expression levels of the studied genes. However, our results indicated that despite overexpression in well-differentiated tumors, the expression levels decreased as the tumors acquired more malignant characteristics. Poorly differentiated tumors expressed lower levels of both *TMPRSS1* and *TMPRSS3*. Notably, both mRNA and protein expression levels were associated with the clinical characteristics of breast cancer: Low expression levels predicted poorer survival and increased risk of distant metastasis compared to high expression levels. Low *TMPRSS1* and *TMPRSS3* expression remained independent factors affecting breast cancer-specific survival in the Cox regression analysis.

These results are consistent with previous studies that reported *TMPRSS1* overexpression in various cancers, especially in prostate cancer [10–14], as well as *TMPRSS3* overexpression in pancreatic and ovarian cancers [16, 17]. We found a notable difference in *TMPRSS1* and *TMPRSS3* mRNA expression between benign samples and grade I malignant tumors in that grade I breast cancer samples expressed considerably higher levels of *TMPRSS1* and *TMPRSS3* than benign samples. This finding supports the theory that hepsin is related to prostate cancer and suggests that hepsin and TMPRSS3 may also play important roles in the early phases of breast carcinogenesis [32, 33]. Our hepsin and TMPRSS3 immunohistochemical staining results correlated with the mRNA expression results. Specifically, samples with more intense cytoplasmic staining were associated with lower tumor grade and stage, and samples with low expression levels were linked to grade III and stage III and IV tumors. Low mRNA expression levels were common in tumors that did not express hormone receptors but that were HER2-positive. In addition, hepsin expression was low in samples with positive nodal status. In the current study, many of the clinical variables that are generally related to advanced breast tumor progression and higher breast cancer mortality rate were linked with low expression levels of the studied genes [34, 35].

Our survival results indicated that low expression of both of the studied genes was an independent prognostic factor in breast cancer. Along with positive nodal status and large tumor size (T3, T4), low mRNA expression remained an independent factor for breast cancer survival. Similarly to our results, Dhanasekaran et al. showed previously that low hepsin protein expression in human prostate cancer samples correlated with poor prostate cancer prognosis [36]. In their study, absent or low hepsin immunostaining was dominant in benign samples, whereas hepsin staining was strong in cancer samples. The strongest hepsin staining was in the precursor lesions of prostate cancer (HG-PIN). Yet among cancer samples, absent or low hepsin expression was associated with prostate-specific antigen (PSA) elevation after radical prostatectomy and large tumor size, indicating poorer survival. In contrast, regarding tumor malignancy, high *TMPRSS1* mRNA expression correlated with advanced tumor stages in prostate cancer [37]. Roemer et al. showed that in renal cell carcinoma, decreased *TMPRSS1* mRNA expression was an independent factor that predicted poorer renal cell carcinoma-specific survival [38]. They suggested that hepsin may be involved in both the early and late development of renal cell carcinoma. However, Betsunoh et al. have observed that hepsin overexpression is associated with poorer renal cell carcinoma survival [39]. In human hepatocellular carcinoma, Chen et al. found that decreased *TMPRSS1* mRNA expression predicted shorter survival time [40]. These studies illustrate variations among the different studies; even so, many of these studies are in agreement with our findings.

In this study we have shown that altered *TMPRSS1* and *TMPRSS3* expressions are associated with the occurrence of relapses and that low *TMPRSS3* mRNA and protein expression are independent factors affecting distant metastasis occurrence. Aberrant expression of TTSPs is associated with tumor invasion and metastasis in various epithelial cancers [6, 41]. Supporting our results, Vasioukhin hypothesized that hepsin may promote metastasis in prostate cancer [32]. This hypothesis suggested that in the initial stages of metastasis, hepsin overexpression might stimulate the invasion of primary tumor cells but, once the cells metastasized, hepsin expression would no longer be essential in distant lesions. We found that distant metastases occurred more frequently during follow-up, in patients with low expression levels of the studied genes in primary tumors. This finding supports the theory that distant metastases are more likely to occur once a certain stage in tumor development is reached, and expression of proteolytic serine proteases is needed from primary tumors to achieve that stage. When local breast cancer relapses and distant metastasis were studied together, low *TMPRSS3* mRNA and protein expression remained independent factors that affected relapse in the Cox regression analyses. On the other hand, based on prostate cancer cell line studies, Srikantan et al. suggested that hepsin overexpression could have antitumorigenic effects and hinted that hepsin might be involved in some sort of positive feedback response [42]. They suggested that decreased hepsin expression could be linked with poor prostate cancer prognosis as exogenously provided hepsin negatively regulated the growth of metastatic prostate cancer cells. However, the first hepsin expression study in MDA-MB-231 and HER18 breast cancer cell lines showed that low hepsin expression levels reduced cell viability and the colony formation rate [14]. Wittig-Blaich et al. showed in a prostate cancer cell line study that the consequences of increased hepsin expression at the cellular level depend on the cell’s microenvironment, and the authors suggested that hepsin overexpression must be spatially and temporally restricted for the efficient development of tumors and metastases [43]. Taken together, these findings support the theory that, depending on the phase of tumorigenesis and metastasis, hepsin expression might either promote or suppress tumors and metastasis.

Based on their proteolytic activity at the cell surface, TTSPs could contribute to tumor progression by affecting initiation of the metastatic process in primary breast cancer tumors. Several substrates for hepsin have been linked to epithelial carcinogenesis, including HGF and uPA. Hepsin and another TTSP, matriptase, efficiently convert inactive pro-HGF to biologically active HGF that, in turn, activates the HGF receptor c-Met [21, 22]. This leads to basement membrane disorganization. Abnormal activation of the HGF/c-Met signaling pathway by aberrant hepsin overexpression is a possible mechanism for the enhancement of tumor progression. In addition, hepsin converts potently pro-uPA into active uPA, which initiates the degradation of ECM by cleaving plasminogen into plasmin [23]. Hepsin may also directly contribute to tumor progression and metastasis by causing defects in cell junctions. Miao et al. showed in human and mouse ovarian cancer cell line studies that hepsin overexpression contributes to ovarian cancer progression via cell membrane interactions with desmosomes [44]. By immunofluorescence they showed that, in addition to cytoplasm hepsin co-localizes with desmosomes at the cell junctions; further, intact desmosomes are required for the membrane localization of hepsin. Supporting these findings, Partanen et al. recently reported that hepsin partially co-localizes with the desmosomal junction protein desmoplakin and, in breast cancer, the two proteins no longer co-localize when lkb1 expression is lost [25]. Notably, loss of lkb1 causes hepsin to relocalize from desmosomes to cytoplasm. Taken together, these studies indicate that the mislocalization and overexpression of hepsin could potentially initiate basement membrane degradation and lead to tumor cell invasion.

The limitations of our study include that *TMPRSS3* expression in cancer has not nearly been studied as extensively as the expression of *TMPRSS1*. More work needs to be done to study the biological role of TMPRSS3 in cancer. Nonetheless, our study presents in a coherent clinical breast cancer sample set that TMPRSS3 is a credible prognostic biomarker. In contrast to our results, a previous study presented that hepsin overexpression was associated with positive nodal status and tumor stage in breast cancer [14]. However, no survival analyses were done in that study and the analysis methods were different. In addition, to validate our results and the prognostic value of the studied genes in a large clinical breast cancer microarray database, we used an online Kaplan-Meier survival analysis tool [31]. Similar to our study, in these analyses the cohorts were divided into two groups according to the median expression of *TMPRSS1* and *TMPRSS3*. Based on the survival curves displayed and the logrank *P* values both low *TMPRSS1* and *TMPRSS3* expression significantly associated with poorer relapse-free survival, overall survival, and distant metastasis-free survival [31]. To sum up, the survival trend was exactly alike compared to our results.

When the treatment data was included in the multivariate survival analyses, low *TMPRSS1* mRNA expression remained an independent factor of poor prognosis in patients who were treated with radiotherapy. It must be highlighted that low *TMPRSS1* expression remained the only significant variable regarding prognosis which excludes for example poor differentiation level of breast cancer cells in these analyses. Furthermore, no significant results were found in patients who were not given any adjuvant therapies. When *TMPRSS1* expression level is higher and epithelial integrity is still rather intact it might be that radiation induced cellular lethality is much more aggressive in breast cancer cells. However, when *TMPRSS1* expression is low and epithelial integrity already damaged it appears that the remaining breast cancer cells are radioresistant leading to these cells surviving which has negative impact on the clinical outcome. Our results indicate that low *TMPRSS1* expression may independently reduce the therapeutic function of radiation yet the specific cellular mechanisms remain unclear. Interestingly, Nakamura et al. showed in an endometrial cancer cell line study that hepsin overexpression resulted in significant cell accumulation at the G2/M phase leading to cell cycle arrest [45]. Cancer cells in general are thought to be the most radiotherapy sensitive exactly at the G2/M phase [46]. These previous studies comply our results even though our significant results are related to mRNA expression and in breast cancer tumor samples. In a previous study of our group we found several *TMPRSS1* and *TMPRSS3* SNP genotypes that associated with survival in patients treated with radiotherapy [26]. We have planned to study in the future the potential associations between our current results with the ones from our SNP study.

The combinations of low expression levels of mRNA and protein were independent factors that predicted poor survival. This suggests that TTSPs are prognostic biomarkers for breast cancer. Matriptase (encoded by the *ST14* gene) is a TTSP that, similar to hepsin, can activate pro-HGF. A recent study showed that hepsin and matriptase are direct pericellular activators of pro-HGF and hypothesized that their suggested ability to autoactivate might be the initial step in HGF/c-Met-mediated basement membrane degradation [47]. Our previous study of matriptase expression in breast cancer resulted in conclusions that were similar to those in the present study in that low matriptase expression was associated with poorer breast cancer survival [27]. However, others have reported the opposite and other studies of matriptase expression in breast cancer have not given consistent results [48]. Notably, since matriptase and hepsin have identical substrates and since both have possible tumor progression and metastasis-promoting activities, further studies of TMPRSS3 are needed to better understand its functions and substrates. Structurally, TMPRSS3 is almost identical to hepsin, and here we have shown that their mRNA and protein expression patterns are very similar in different phases of breast carcinogenesis and correlate with breast cancer prognosis. Co-expression of these proteolytic serine proteases could enhance their effects on tumor cell invasion and metastasis.

## Conclusions

In closing, this study expands our knowledge of the biological processes behind breast cancer by investigating hepsin and TMPRSS3 expression in human breast tumors. Low mRNA and protein expression levels of the studied TTSPs were prognostic markers of poor survival in breast cancer. Furthermore, low *TMPRSS1* mRNA expression is an independent marker of poor clinical outcome in patients treated with radiotherapy. We think that our results give emphasis to the role of altered expression of hepsin and TMPRSS3 in promoting breast tumor progression and metastasis as their role in breast cancer is still rather unexplored. The results showed that both TTSPs have potential as prognostic biomarkers.

## Additional files

Additional file 1: Table S1. Significant clinical variables associated with hepsin and TMPRSS3 protein expression.
Additional file 2: Figure S1. The studied A, TMPRSS1 and B, TMPRSS3 mRNA expression levels in benign breast tumor samples and in malignant tumors grouped by tumor grade. In benign tumors, the mRNA expression levels are lower compared to grade I tumors. Tumor grade is inversely associated with *TMPRSS1* and *TMPRSS3* mRNA expression levels. In grade I tumors, the cancer cells were well differentiated, in grade II tumors, the cells were moderately differentiated, and in grade III tumors, the cancer cells were poorly differentiated or undifferentiated. **P* < 0.005 mRNA expression in benign tumors versus grade I tumors and grade I tumors versus grade III tumors.
Additional file 3: Figure S2. Breast cancer relapse-free survival compared to TMPRSS3 expression levels in Kaplan-Meier survival analysis and in Cox regression multivariate analysis. Both local and distant recurrences were taken into account. Patients were divided into high and low expression groups relative to the median expression values. Low expression levels of A, TMPRSS3 mRNA (median survival time 7.73 years) and B, TMPRSS3 protein (median survival time 8.84 years) associated with relapses occurring more frequently. Low expression levels of C, TMPRSS3 mRNA (median survival time 9.10 years) and D, TMPRSS3 protein (median survival time 7.66 years) expression remained independent prognostic factors of more frequent occurrence of breast cancer relapse. In addition to expression levels, tumor grade, nodal status, tumor size, ER status, PR status, and histologic type were included in the multivariate analyses.
Additional file 4: Figure S3. Breast cancer distant metastasis-free survival in Kaplan-Meier survival analysis. Patients were divided into high and low expression groups relative to the median expression values. Low expression levels of A, TMPRSS1 mRNA (median survival time 7.66 years); B, TMPRSS3 mRNA (median survival time 7.78 years); and C, TMPRSS3 protein (median survival time 9.13 years) associated with distant metastasis occurring more frequently.
Additional file 5: Table S2. Significant clinical variables associated with combined TMPRSS1-TMPRSS3 mRNA.
Additional file 6: Figure S4. Breast cancer survival and distant metastasis-free survival compared to combined mRNA and protein expression variables in Kaplan-Meier survival analysis. The ‘combined low expression’ groups of A; C, TMPRSS1 and TMPRSS3 mRNA (A, median survival time 9.84 years; C, median survival time 7.96 years); and B; D, hepsin and TMPRSS3 protein (B, median survival time 10.94 years; D, median survival time 9.13 years) expression associated with poorer breast cancer specific survival (A, B) and with distant metastasis (C, D) occurring more frequently.
Additional file 7: Figure S5. The prognostic value of low *TMPRSS1* and *TMPRSS3* mRNA expression levels in breast cancer was validated in a public gene expression dataset. The results obtained from the online Kaplan-Meier plotter analysis tool presented that both low *TMPRSS1* and *TMPRSS3* expression were significantly associated with poorer relapse-free survival (*TMPRSS1*, A; *TMPRSS3*, B), overall survival (*TMPRSS1*, C; *TMPRSS3*, D), and distant metastasis-free survival in breast cancer (*TMPRSS1*, E; *TMPRSS3*, F). Patients were divided into high and low expression groups relative to the median expression values.

## Abbreviations

TTSPs: Type II transmembrane serine proteases
TMPRSS1/3: Transmembrane protease, serine 1/3
ECM: Extracellular matrix, HGF, hepatocyte growth factor
uPA: Urokinase-type plasminogen activator
lkb1: Liver kinase B1
ln-332: Laminin-332
KBCP: Kuopio Breast Cancer Project
PPIA: Peptidylprolyl isomerase A
TMA: Tissue microarray
DAB: Diaminobenzidine tetrahydrochloride
OR: Odds ratio
CI: Confidence interval
HR: Hazards ratio
ER: Estrogen receptor
PR: Progesterone receptor
HER2: Human epidermal growth factor receptor 2
HG-PIN: High-grade prostatic intraepithelial neoplasia
PSA: Prostate-specific antigen
