# Supplementary material for: Low expression levels of hepsin and TMPRSS3 are associated with poor breast cancer survival
Source: BMC Cancer. 2015 May 27;15:431. doi: 10.1186/s12885-015-1440-5 (PMC4445813; doi:10.1186/s12885-015-1440-5)
Supplement: Additional file 1: Table S1. — Significant clinical variables associated with hepsin and TMPRSS3 protein expression. [file 12885_2015_1440_MOESM1_ESM.pdf]

Supplementary Table S1. Significant clinical variables associated with hepsin and TMPRSS3 protein expression

Clinical variable (%)	Protein expression[a]			
	**Low**	**Moderate/high, Ref.**	***P*** [b]	**OR (95 % CI)**[c]
**Hepsin**				
Tumor grade			**0.0004**[b]	
I	28 (32.2)	59 (67.8)		Ref.
II	76 (46.9)	86 (53.1)	**0.025**	**1.862 (1.079-3.213)**
III	64 (61.0)	41 (39.0)	**0.00009**	**3.289 (1.811-5.973)**
Tumor stage			**0.015**[b]	
I	48 (39.7)	73 (60.3)		Ref.
II	91 (49.5)	93 (50.5)	0.094	1.488 (0.935-2.369)
III, IV	29 (64.4)	16 (35.6)	**0.005**	**2.757 (1.354-5.611)**
Tumor size			**0.025**[b]	
T1	72 (41.6)	101 (58.4)		Ref.
T2	86 (53.8)	74 (46.2)	**0.027**	**1.630 (1.057-2.515)**
T3, T4	21 (61.8)	13 (38.2)	**0.034**	**2.266 (1.065-4.821)**
Nodal status			**0.010**[b]	
Negative	82 (41.6)	115 (58.4)		Ref.
Positive	86 (55.5)	69 (44.5)	**0.010**	**1.748 (1.143-2.674)**
**TMPRSS3**				
Tumor grade			**0.0000008**[b]	
I	29 (33.0)	59 (67.0)		Ref.
II	85 (52.8)	76 (47.2)	**0.002**	**2.341 (1.364-4.018)**
III	75 (70.8)	31 (29.2)	**0.0000002**	**5.006 (2.721-9.209)**
Tumor stage			**0.007**[b]	
I	51 (41.8)	71 (58.2)		Ref.
II	109 (59.2)	75 (40.8)	**0.002**	**2.109 (1.328-3.348)**
III, IV	27 (60.0)	18 (40.0)	**0.028**	**2.176 (1.086-4.361)**
Tumor size			**0.002**[b]	
T1	79 (45.4)	95 (54.6)		Ref.
T2	102 (63.8)	58 (36.2)	**0.00083**	**2.115 (1.363-3.281)**
T3, T4	16 (45.7)	19 (54.3)	0.973	1.013 (0.489-2.099)
PR status			**0.022**[b]	
Negative	87 (61.7)	54 (38.3)	**0.017**	**1.679 (1.098-2.568)**
Positive	103 (48.8)	108 (51.2)		Ref.
ER/PR/HER2 status			**0.022**[b]	
Triple-negative	30 (69.8)	13 (30.2)	**0.022**	**2.244 (1.125-4.477)**
Non-triple-negative	146 (50.7)	142 (49.3)		Ref.
Histological type			0.060[b]	
Ductal	131 (55.3)	106 (44.7)	**0.031**	**1.760 (1.053-2.942)**
Lobular	43 (59.7)	29 (40.3)	**0.024**	**2.112 (1.105-4.037)**
Other malignant	26 (40.6)	38 (59.4)		Ref.

NS, not significant; Ref., reference category in the logistic regression analysis

[a]The median value of protein expression was used in the analyses.

[b]*P* value assessed by Fisher's exact test. Other *P* values from logistic regression analysis.

[c]OR and 95% CI values for association from logistic regression analysis.
