# Supplementary figures and images for: Low expression levels of hepsin and TMPRSS3 are associated with poor breast cancer survival
Source: BMC Cancer. 2015 May 27;15:431. doi: 10.1186/s12885-015-1440-5 (PMC4445813; doi:10.1186/s12885-015-1440-5)

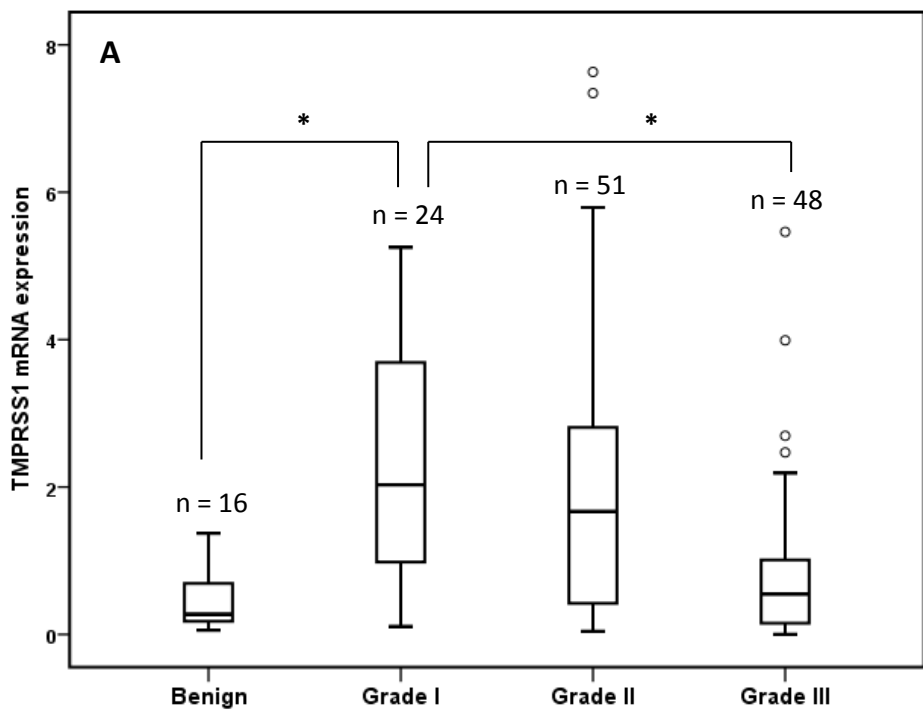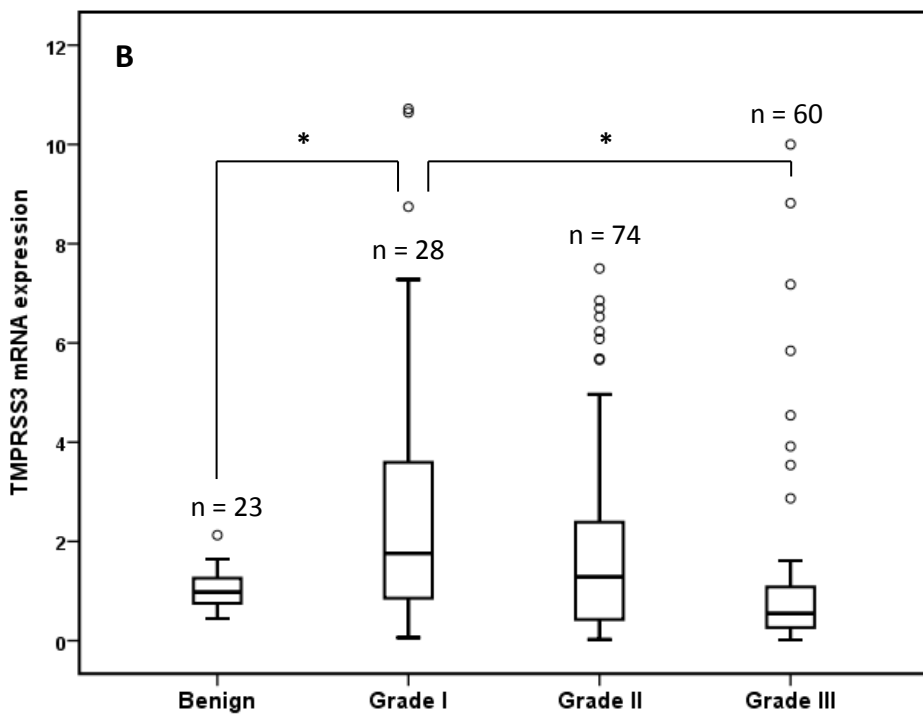

Supplement: Additional file 2: Figure S1. — The studied A, TMPRSS1 and B, TMPRSS3 mRNA expression levels in benign breast tumor samples and in malignant tumors grouped by tumor grade. In benign tumors, the mRNA expression levels are lower compared to grade I tumors. Tumor grade is inversely associated with TMPRSS1 and TMPRSS3 mRNA expression levels. In grade I tumors, the cancer cells were well differentiated, in grade II tumors, the cells were moderately differentiated, and in grade III tumors, the cancer cells were poorly differentiated or undifferentiated. *P < 0.005 mRNA expression in benign tumors versus grade I tumors and grade I tumors versus grade III tumors. [file 12885_2015_1440_MOESM2_ESM.pdf]

Supplementary Figure S2

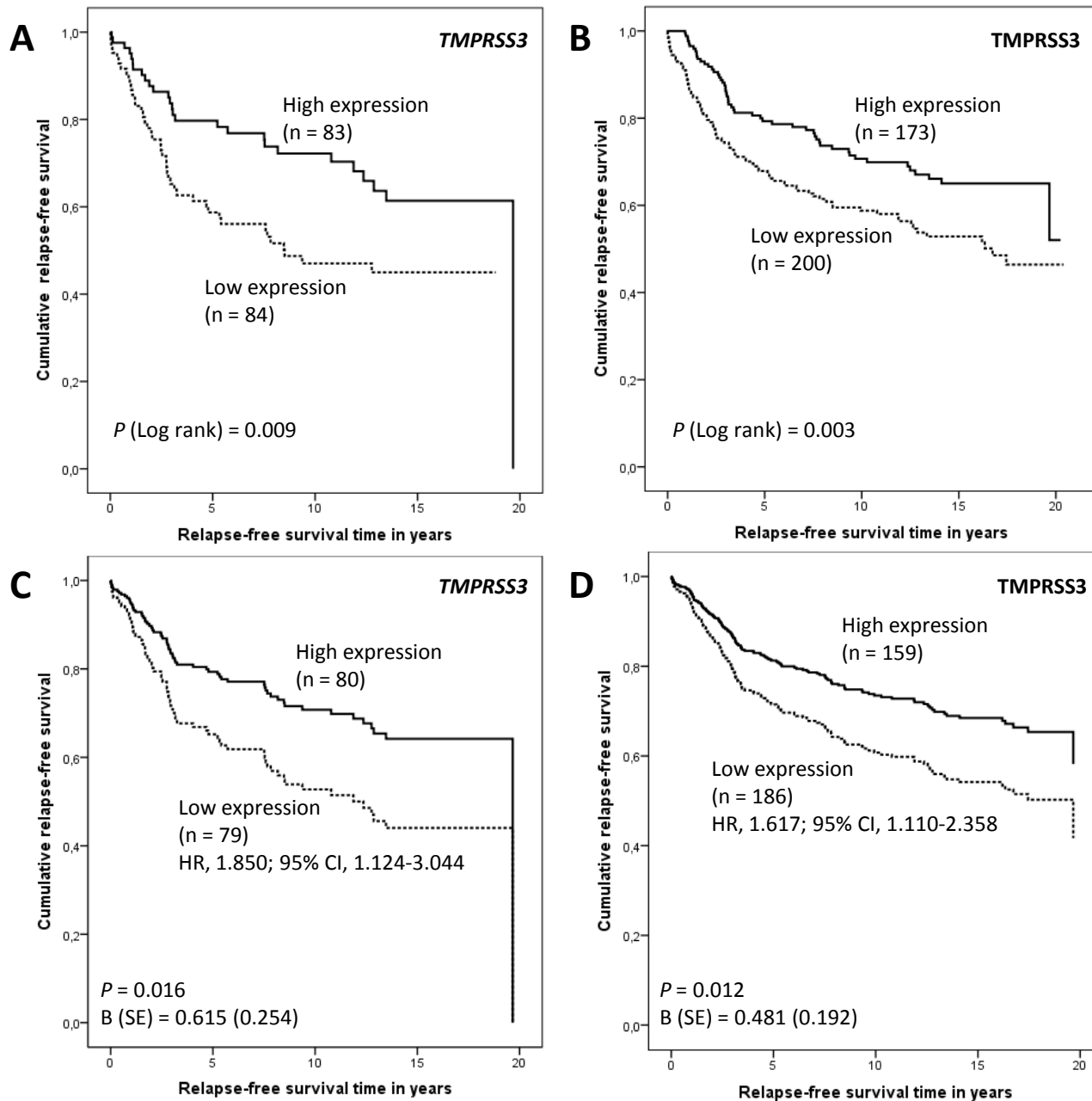

Supplement: Additional file 3: Figure S2. — Breast cancer relapse-free survival compared to TMPRSS3 expression levels in Kaplan-Meier survival analysis and in Cox regression multivariate analysis. Both local and distant recurrences were taken into account. Patients were divided into high and low expression groups relative to the median expression values. Low expression levels of A, TMPRSS3 mRNA (median survival time 7.73 years) and B, TMPRSS3 protein (median survival time 8.84 years) associated with relapses occurring more frequently. Low expression levels of C, TMPRSS3 mRNA (median survival time 9.10 years) and D, TMPRSS3 protein (median survival time 7.66 years) expression remained independent prognostic factors of more frequent occurrence of breast cancer relapse. In addition to expression levels, tumor grade, nodal status, tumor size, ER status, PR status, and histologic type were included in the multivariate analyses. [file 12885_2015_1440_MOESM3_ESM.pdf]

Supplementary Figure S3

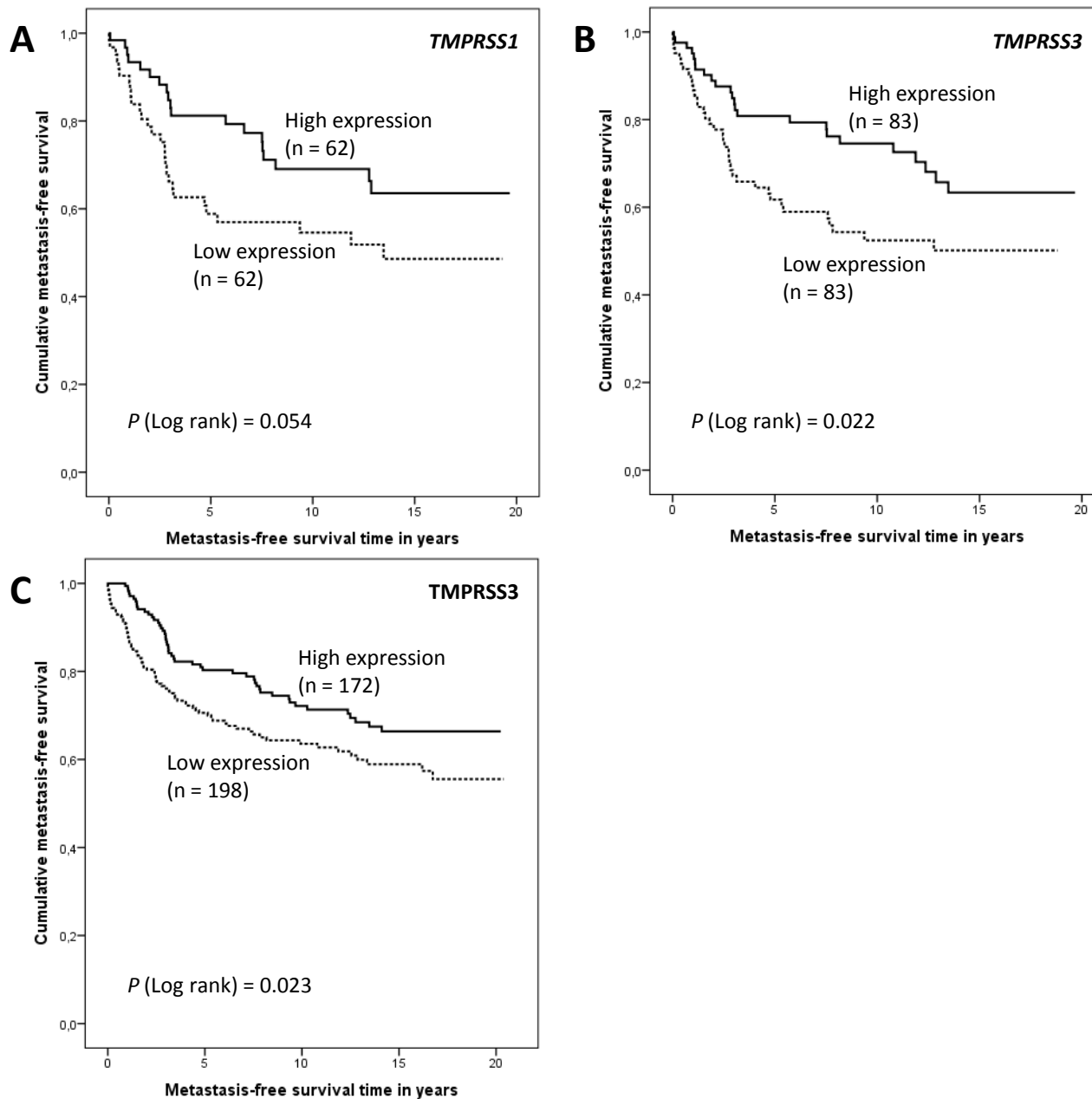

Supplement: Additional file 4: Figure S3. — Breast cancer distant metastasis-free survival in Kaplan-Meier survival analysis. Patients were divided into high and low expression groups relative to the median expression values. Low expression levels of A, TMPRSS1 mRNA (median survival time 7.66 years); B, TMPRSS3 mRNA (median survival time 7.78 years); and C, TMPRSS3 protein (median survival time 9.13 years) associated with distant metastasis occurring more frequently. [file 12885_2015_1440_MOESM4_ESM.pdf]

## Supplementary Figure S4

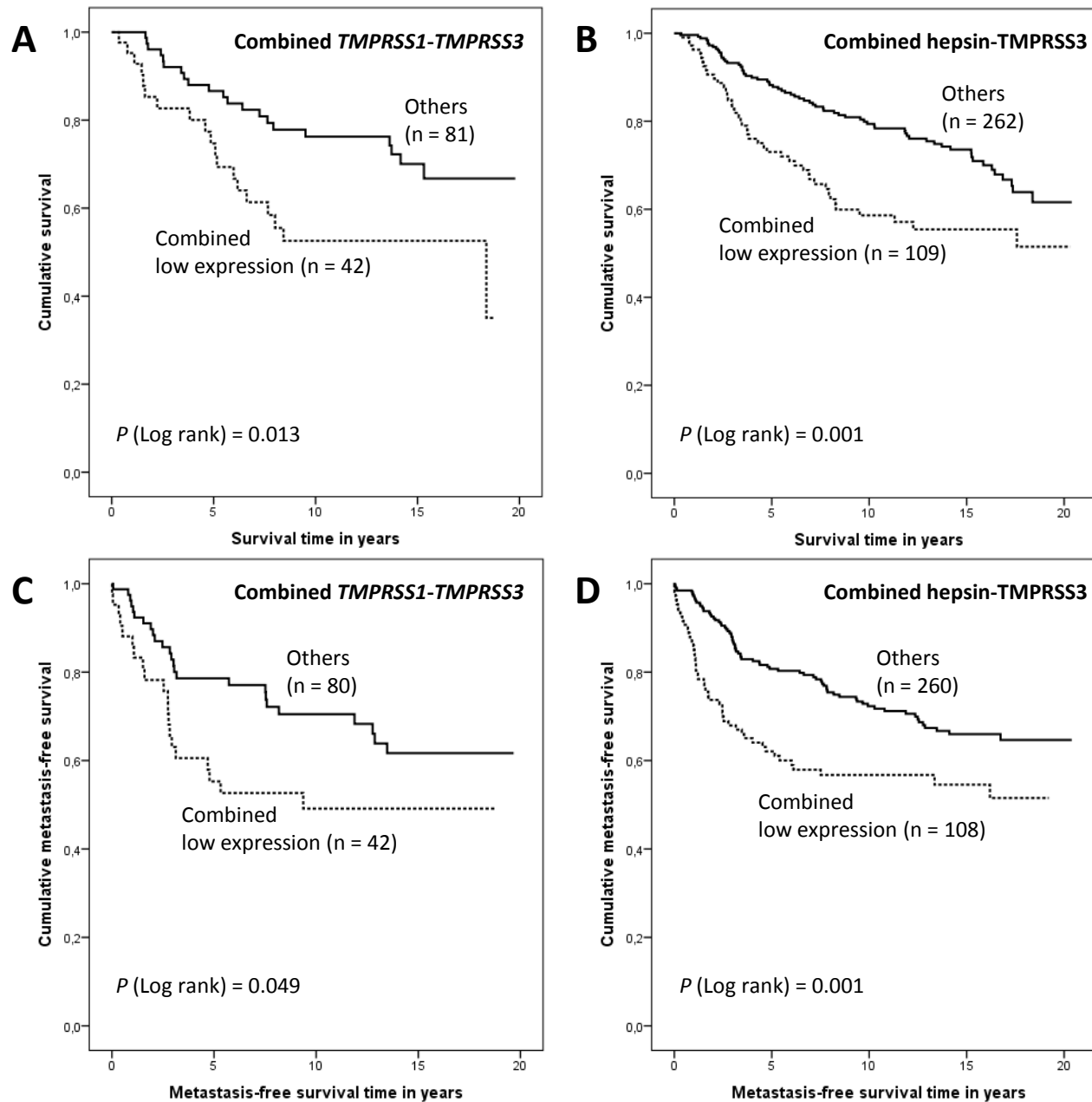

Supplement: Additional file 6: Figure S4. — Breast cancer survival and distant metastasis-free survival compared to combined mRNA and protein expression variables in Kaplan-Meier survival analysis. The ‘combined low expression’ groups of A; C, TMPRSS1 and TMPRSS3 mRNA (A, median survival time 9.84 years; C, median survival time 7.96 years); and B; D, hepsin and TMPRSS3 protein (B, median survival time 10.94 years; D, median survival time 9.13 years) expression associated with poorer breast cancer specific survival (A, B) and with distant metastasis (C, D) occurring more frequently. [file 12885_2015_1440_MOESM6_ESM.pdf]

Supplementary Figure S5

Source: <http://kmplot.com/analysis/index.php?p=service&cancer=breast>

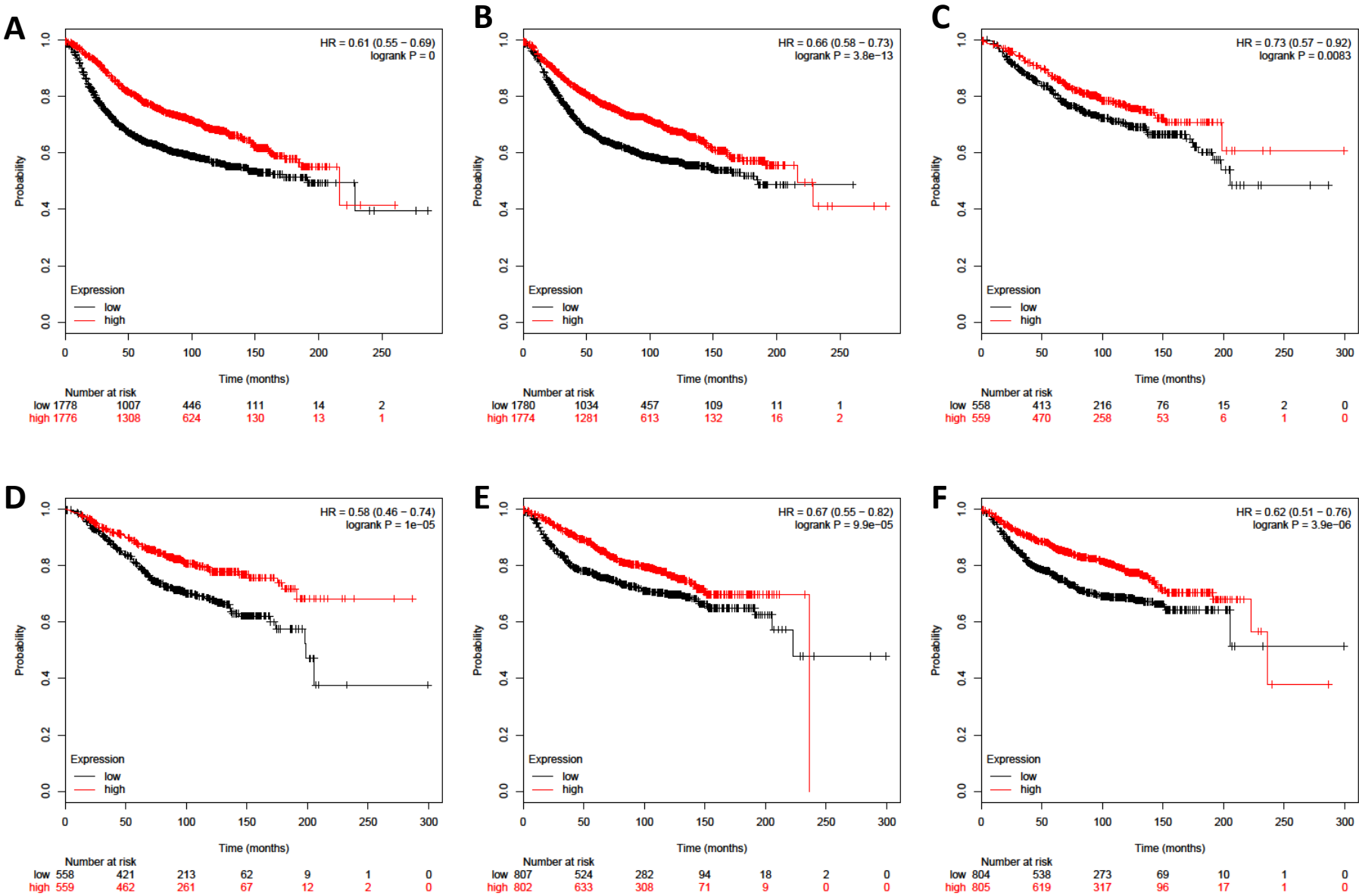

Supplement: Additional file 7: Figure S5. — The prognostic value of low TMPRSS1 and TMPRSS3 mRNA expression levels in breast cancer was validated in a public gene expression dataset. The results obtained from the online Kaplan-Meier plotter analysis tool presented that both low TMPRSS1 and TMPRSS3 expression were significantly associated with poorer relapse-free survival (TMPRSS1, A; TMPRSS3, B), overall survival (TMPRSS1, C; TMPRSS3, D), and distant metastasis-free survival in breast cancer (TMPRSS1, E; TMPRSS3, F). Patients were divided into high and low expression groups relative to the median expression values. [file 12885_2015_1440_MOESM7_ESM.pdf]
