# Supplementary material for: Low expression levels of hepsin and TMPRSS3 are associated with poor breast cancer survival
Source: BMC Cancer. 2015 May 27;15:431. doi: 10.1186/s12885-015-1440-5 (PMC4445813; doi:10.1186/s12885-015-1440-5)
Supplement: Additional file 5: Table S2. — Significant clinical variables associated with combined TMPRSS1-TMPRSS3 mRNA. [file 12885_2015_1440_MOESM5_ESM.pdf]

Supplementary Table S2. Significant clinical variables associated with combined *TMPRSS1-TMPRSS3* mRNA and protein expression

**Clinical variable**	**Low**	**Others, Ref.**	***P*** [b]	**OR (95 % CI)** [c]
**Combination of mRNA expression[a] (%)**				
Tumor grade			**0.00018[b]**	
I, II	15 (20.5)	58 (79.5)		Ref.
III	26 (54.2)	22 (45.8)	**0.0002**	**4.570 (2.047 -10.200)**
ER status			**0.001[b]**	
Negative	22 (55.0)	18 (45.0)	**0.001**	**3.988 (1.779 - 8.944)**
Positive	19 (23.5)	62 (76.5)		Ref.
PR status			**0.020[b]**	
Negative	25 (45.5)	30 (54.5)	**0.015**	**2.604 (1.201 -5.645)**
Positive	16 (24.2)	50 (75.8)		Ref.
HER2 status			**0.027[b]**	
Negative	27 (27.6)	71 (72.4)		Ref.
Positive	10 (55.6)	8 (44.4)	**0.024**	**3.287 (1.174 - 9.206)**
**Combination of protein expression[a] (%)**				
Tumor grade			**0.0000006[b]**	
I	10 (11.5)	77 (88.5)		Ref.
II	43 (26.7)	118 (73.3)	**0.007**	**2.806 (1.331-5.914)**
III	48 (45.7)	57 (54.3)	**0.0000015**	**6.484 (3.025-13.900)**
Tumor stage			**0.0037[b]**	
I	22 (18.3)	98 (81.7)		Ref.
II	61 (33.2)	123 (66.8)	**0.005**	**2.209 (1.268-3.848)**
III, IV	18 (40.0)	27 (60.0)	**0.005**	**2.970 (1.396-6.316)**
Tumor size			**0.004[b]**	
T1	37 (21.5)	135 (78.5)		Ref.
T2	60 (37.5)	100 (62.5)	**0.002**	**2.189 (1.349-3.554)**
T3, T4	12 (35.3)	22 (64.7)	0.088	1.990 (0.902-4.393)
Nodal status			**0.0089[b]**	
Negative	45 (23.0)	151 (77.0)		Ref.
Positive	56 (36.1)	99 (63.9)	**0.007**	**1.898 (1.190-3.028)**
PR status			**0.031[b]**	
Negative	50 (35.7)	90 (64.3)	**0.026**	**1.699 (1.066-2.708)**
Positive	52 (24.6)	159 (75.4)		Ref.
Histological type			**0.012[b]**	
Ductal	76 (32.2)	160 (67.8)	**0.007**	**2.850 (1.337-6.074)**
Lobular	24 (33.3)	48 (66.7)	**0.012**	**3.000 (1.271-7.083)**
Other malignant	9 (14.3)	54 (85.7)		Ref.

Ref., reference category in the logistic regression analysis

[a]The 'combined low group' included all cases with low expression levels of both genes.

[b]*P* value assessed by Fisher's exact test. Other *P* values from the logistic regression analysis.

[c]OR and 95% CI values for association from the logistic regression analysis.
